# Helicobacter cinaedi bacteraemia secondary to enterocolitis in an immunocompetent patient

**DOI:** 10.1186/s13099-021-00422-8

**Published:** 2021-04-23

**Authors:** Sofie Larsen Rasmussen, Iben Ørsted, Irene Harder Tarpgaard, Hans Linde Nielsen

**Affiliations:** 1 Department of Clinical Microbiology, Aalborg University Hospital, Hobrovej 18, 9000 Aalborg, Denmark; 2 Department of Infectious Diseases, Aalborg University Hospital, Hobrovej 18, 9000 Aalborg, Denmark; 3 Department of Clinical Medicine, Aalborg University, Aalborg, Denmark

**Keywords:** *Helicobacter cinaedi*, Bacteraemia, Gastroenteritis, Enterocolitis, MLST, SNP-analysis

## Abstract

**Background:**

*Helicobacter cinaedi* are motile, gram-negative spiral rods with a natural reservoir in the intestinal tract of hamsters and rhesus monkeys. In humans, *H. cinaedi* has been reported in different human infections like fever, abdominal pain, gastroenteritis, proctitis, diarrhoea, erysipelas, cellulitis, arthritis, and neonatal meningitis typically diagnosed by positive blood cultures. Even though *H. cinaedi* has been detected from human blood and stool the entry of *H. cinaedi* into the blood stream was undocumented until quite recently. The use of pulse-field gel electrophoresis (PFGE) demonstrated that stool- and blood-derived *H. cinaedi* strains were consistent.

**Case presentation:**

Here, we describe a rare Danish case of *H. cinaedi* bacteraemia in an immunocompetent 44-year-old male with diarrhoea. We isolated *H. cinaedi* from a blood culture taken at admission, and from a FecalSwab taken at day six despite ongoing antibiotic therapy. Next, we made a genetic comparison of both isolates by use of Multi-locus sequence typing (MLST)- and Single nucleotide polymorphism (SNP)-analysis. The two isolates were identical with zero SNPs and by use of MLST the isolate was identified as a novel ST20, confirming previous data of the intestinal tract as a route of *H. cinaedi* bacteraemia. The results of our AST showed a resistance pattern with higher MICs for ciprofloxacin and clarithromycin than for ampicillin, amoxicillin, gentamicin, and imipenem. The patient was cured with targeted therapy with pivampicillin; however, the primary source of transmission was unknown.

**Conclusions:**

In conclusion, this case of *H. cinaedi* bacteraemia secondary to enterocolitis in an immunocompetent patient provide clear evidence that one route of infection occurs through translocation from the intestinal tract to the bloodstream. *Helicobacter cinaedi* from blood and faeces were identical with a novel ST20, resistant to ciprofloxacin and clarithromycin however, the patient was cured with oral pivampicillin.

## Background

*Helicobacter cinaedi* was originally described as *Campylobacter cinaedi* by Totten et al. in 1985 however, six year later it was reclassified into genus *Helicobacter* by Vandamme et al. [1, 2]. These organisms are motile, gram-negative spiral rods which have been isolated from different domestic animals including dogs, cats and foxes [3]. However, the only natural reservoir of *H. cinaedi* is the intestinal tract of hamsters and rhesus monkeys [3, 4].

In humans, *H. cinaedi* was first isolated from the rectal cultures of HIV-positive, homosexual men with proctitis, proctocolitis, and enteritis [5]. It was initially considered an opportunistic pathogen that caused disease in immunocompromised patients however in recent years infections in immunocompetent patients have also been reported [6]. Most *H. cinaedi* infections with symptoms such as fever, abdominal pain, gastroenteritis, proctitis, diarrhoea, erysipelas, cellulitis, arthritis and neonatal meningitis have been identified by positive blood cultures [5–8].

Even though *H. cinaedi* was detected in both blood and stool cultures the entry of *H. cinaedi* into the blood stream was undocumented for decades. However, in 2018 Araoka et al. demonstrated by use of pulse-field gel electrophoresis (PFGE) that stool- and blood-derived *H. cinaedi* strains were consistent in nine Japanese patients giving first evidence of translocation from the intestinal tract as one of the routes that leads to *H. cinaedi* bacteraemia [9].

Here, we describe a rare Danish case of *H. cinaedi* bacteraemia in an immunocompetent patient with diarrhoea. We isolated *H. cinaedi* from blood and faeces and made a genetic comparison of the isolates by use of Multi-locus sequence typing (MLST)- and Single nucleotide polymorphism (SNP)-analysis and found that they were identical, confirming previous data of the intestinal tract as a route of *H. cinaedi* bacteraemia [9].

## Case presentation

A 44-year-old male with a family history of polycystic kidney disease though otherwise healthy with no medical record was admitted to Aalborg University Hospital on suspicion of chronic kidney disease. The patient presented with nausea and diarrhoea with five lose watery stools a day for two weeks, and a sudden onset of fever, chills, flank and lower back pain, and dark urine. He did not report any bloody diarrhoea, nor dysuria, polyuria, urgency or haematuria. His temperature was 39.8 °C however, the physical examination and his vital parameters were normal. Laboratory findings showed leucocytosis with a WBC-count of 17.5 × 10^9^/L, an elevated CRP of 90 mg/L, and a mild hyponatremia (p-sodium: 136mmol/L) and hypokalaemia (p-potassium: 3.4 mmol/L) however, there was a normal p-creatinine of 80 µmol/L. Urine and blood culture was obtained, and on suspicion of urinary tract infection standard empiric antibiotic therapy was initiated with intravenous ampicillin 2 g every 6 h, and gentamicin 5 mg/kg once daily. After two days the patient was afebrile nevertheless, CRP was further elevated to 301. Therefore, the antibiotic therapy was changed to intravenous mecillinam (amdinocillin) 1 g every 8 h. The chest x-ray was normal however, ultrasound showed multiple cysts in the liver and in both kidneys however, no sign of hydronephrosis.

Urine culture was negative. On day six there was a positive blood culture with growth of Gram-negative spiral-shaped rods and on preliminary suspicion of *Campylobacter* bacteraemia oral roxithromycin 150 mg twice daily was initiated. In addition, a stool culture was sent to the laboratory. Seven days after hospitalization he was afebrile, CRP had dropped to 133 mg/L. The diarrhoea symptoms were declining, and still he had no urinary symptoms, so he was discharged with oral pivmecillinam 400 mg every 8 h and roxithromycin. At day 10 the patient was seen in an outpatient setting and he reported ongoing diarrhoea and with a simultaneous identification of *H. cinaedi* in blood and stools antibiotic therapy was changed to oral pivampicillin 500 mg every 8 h for seven days. At day 17, he no longer had diarrhoea and CRP was normalised at 5.7 mg/L. The patient owned a dog, and had no close contact to other animals and was immunocompetent.

## Investigations

A standard blood culture was obtained upon submission (Two BD BACTEC™ Plus Aerobic medium and one BD BACTEC™ Lytic Anaerobic medium glass culture vials) incubated in the BACTEC FX Top instrument (Becton Dickinson AB, Stockholm, Sweden). After six days of incubation there was growth in the two aerobic bottles (Time to detection (TTD): 141.3 and 143.6 h, respectively) of motile, Gram-negative, spiral-shaped rods. Sub-cultivation was performed on 5 % yeast-enriched horse blood agar plates (SSI Diagnostica, Hillerød, Denmark) at 37 °C in a hydrogen-enriched microaerobic atmosphere (6% O_2_, 6% CO_2_, 6% H_2_, and 82% N_2_) and examined after 48 h. The isolate was urease negative though oxidase and catalase positive, and final identification of *H. cinaedi* was performed by use of the matrix-assisted laser desorption ionization–time of flight (MALDI Biotyper 3.1, Bruker Daltonics Microflex LT, MBT 6903 MSP Library) with a score of 2.060.

No clinical breakpoints exist for *H. cinaedi*, however, antibiotic susceptibility testing (AST) was performed by use of McFarland standard 1.0 on Mueller-Hinton agar for fastidious organisms (MH supplemented with 5% defibrinated horse blood and 20 mg/L β-NAD) and Etest (BioMérieux, Marcy l’Etoile, France) in a microaerobic atmosphere. The isolate showed low minimum inhibitory concentrations (MICs) to ampicillin (MIC: 2 mg/L), amoxicillin (MIC: 4 mg/L), imipenem (MIC: 0.032 mg/L) and gentamicin (MIC: 0.25 mg/L) and resistant to ciprofloxacin (MIC: > 32 mg/L) and clarithromycin (MIC: > 256 mg/L). For mecillinam (no etest available) we included disc diffusion (disc content, 10 µg), showing an inhibition zone of 18 mm, however, we were unable to interpret this result.

After the positive blood culture on day 6 a FecalSwab (COPAN ITALIA, Brescia, Italy) was sent to the laboratory. The sample was negative for *Aeromonas*, *Campylobacter*, *Salmonella*, *Shigella*, *Vibrio*, *Yersinia enterocolitica* by routine culture methods and *Clostridioides difficile* toxins by use of the Xpert *C. difficile* BT (Cepheid). However, 0.5 ml liquid media from the FecalSwab were incubated by use of a polycarbonate filter method on a 5% yeast-enriched blood agar plate (SSI Diagnostica, Hillerød, Denmark) as described elsewhere [10]. After 48 h at 37 °C in a hydrogen-enriched microaerobic atmosphere, there was growth of *H. cinaedi*, identified with a MALDI log-score of 2.080.

Next, we performed genome sequencing of both isolates to investigate whether the *H. cinaedi* in faeces and blood were identical. We used the Illumina MiSeq instrument producing 2 × 300-bp paired-end reads by using Nextera XT library preparation kit (Illumina Denmark ApS, Copenhagen, Denmark). Reads were assembled using CLC Genomics Workbench (version 11) (QIAGEN Bioinformatics, Aarhus, Denmark) into contigs (n = 74 + n = 72 ≥ 1 kb, respectively), N50 (89,940 + 96,798, respectively), total sequence length 2,406,086 + 2,403,138 bp, respectively, and with a G + C content of 38.4 %. To subtype our strains and identify sequence type (ST) we used the *H. cinaedi* Multilocus Sequence Typing (MLST) database [11]. Both isolates were classified as a novel ST20 (allelic profile, 23S_rRNA: 5, *ppa*: 7, *aspA*: 5, *aroE*: 7, *atpA*: 3, *tkt*: 5, *cdtB*: 3) (The blood isolate, AAUH 190,263, was uploaded to: https://pubmlst.org/). Next, we used the CSI Phylogeny v1.4 pipeline (with default-settings) for SNP-calling between isolates [12], which showed 0 SNPs, giving clear evidence of identical strains and a translocation of *H. cinaedi* from the intestinal tract into the bloodstream.

## Discussion and conclusions

This is the first Danish case of *H. cinaedi* bacteraemia with documented evidence of translocation from the intestinal tract in an immunocompetent patient. We isolated *H. cinaedi* from blood in two aerobic BACTEC bottles after six days of incubation, and faeces by use of the polycarbonate filtration technique. By use of MLST and SNP-analyses we identified the two isolates as a single (zero SNPs) ST20. Araoka et al. evaluated the TTD for *H. cinaedi* in the BACTEC system and found median time of 5 days (range, 2 to 12 days), and all *H. cinaedi* strains (126 positive sets) were detected in aerobic bottles only [7]. The blood cultures in our laboratory are routinely incubated for 7 days, which theoretically should be sufficient to find 87% of ‘true’ *H. cinaedi*-positive blood cultures [7]. In comparison, Kawamura et al. also reported positive results with the VersaTREK systems providing excellent growth ability for *H. cinaedi* [8] however, to our knowledge the VersaTREK system is not available in Denmark.

*Helicobacter cinaedi* infection seems very uncommon in Denmark, with only two previous cases: One case with blood stream infection secondary to cellulitis, and one case with septic arthritis, respectively [13, 14]. Nielsen et al. investigated 5.963 diarrheic stool samples from 4.094 patients and identified two isolates of *H. cinaedi* by use of the polycarbonate filter method [10]. Thus, *H. cinaedi* is uncommon in Danish stool samples. In contrast, *H. cinaedi* has been isolated from diarrheic stool samples and blood cultures of paediatric patients in South Africa [15], and it has been reported that *H. cinaedi* constitutes approximately 2.2% of positive blood cultures in Japan [7]. Recurrent bacteraemia has also been described with cellulitis as the most frequent clinical symptom [16–19]. The cause of the substantial difference in occurrence of *H. cinaedi* infection between Denmark and Japan is unknown.

Through genome sequencing we confirmed that the isolates of *H. cinaedi* from stool and blood were identical by MLST-profile and zero SNPs. Previously, MLST were used to identify *H. cinaedi* STs causing nosocomial outbreaks in Japanese hospitals, whereas some isolates were non-typeable by Pulsefield gel electrophoresis PFGE [11]. In the MLST-database the majority of *H. cinaedi* isolates (n = 70 per 2021-02-10) are from Japan (89%), USA or Australia, so the designation of a ‘novel’ ST is not surprising. Our isolate is also the single European *H. cinaedi* isolate in the database.

Until recently, the entrance of *H. cinaedi* into the blood stream were more or less undocumented and Kawamura et al. stated the importance of pursuing the route of infection [8]. In 2018, Araoka et al. described PFGE typing to confirm clonality of the faeces and blood isolates from nine immunocompromised patients with fever and cellulitis, delivering the first evidence of bacterial translocation from the intestinal tract as a route of *H. cinaedi* bacteraemia [9]. Our patient did not show any signs of cellulitis in addition to diarrhoea, nor was he immunocompromised, whereas the nine patients described by Araoka et al. were treated with either steroids or anticancer chemotherapy [9]. Interestingly, the same study group identified anticancer chemotherapy and systemic steroids as independent risk factors for recurrent *H. cinaedi* bacteraemia [16], a condition that apparently seems rare outside of Japan. Previous case reports have also described hepatic and kidney cyst infection with bacteraemia caused *H. cinaedi* [20–22], however the radiological imaging showed no sign of infection in the cysts in the presented case.

*Helicobacter cinaedi* is not known to normally colonize the intestines of humans, and it has been proposed that infection might develop through zoonotic transmission [8], or through nosocomial spread among immunocompromised patients during hospitalization [23, 24]. Our patient owned a dog, and had no close contact with other animals, so the primary source of transmission remains unknown. Kiehlbauch et al. showed that *H. cinaedi* isolates from human, dog, and hamster formed distinct ribotype pattern [25], and two decades later Kawamura et al. found that isolates from dogs which had been identified as *H. cinaedi* were different from human *H. cinaedi* isolates by whole-genome in silico DNA similarities and therefore re-classified as the new *Helicobacter canicola* [26].

There is no clinical breakpoints nor recommended guideline for the choice or duration of antibiotic therapy for *H. cinaedi* infection. Recently, Nukui et al. suggested a two-week antibiotic therapy to prevent recurrence of *H cinaedi* bacteremia [27]. The results of our AST were in coherence with previously reported resistance patterns of *H. cinaedi* with higher MICs for ciprofloxacin and clarithromycin than for ampicillin, amoxicillin, gentamicin, and imipenem [8, 27]. After admission, the patient responded on empiric therapy with ampicillin and gentamicin and became afebrile. However, the empiric antibiotic regime was later changed to intravenous mecillinam, and despite this therapy *H. cinaedi* could be isolated from a FecalSwab taken six days after admission. Pivmecillinam and roxithromycin had also no clinical effect as diarrheal symptoms resided. However, a change to pivampicillin stopped the diarrheal symptoms and CRP normalized, and to our knowledge the patient has not been hospitalized since.

In conclusion, this case of *H. cinaedi* bacteraemia secondary to enterocolitis in an immunocompetent patient provide clear evidence that one route of infection occurs through translocation from the intestinal tract to the bloodstream. *Helicobacter cinaedi* from blood and faeces were identical with a ‘novel’ ST20, resistant to ciprofloxacin and clarithromycin however, the patient was cured with oral pivampicillin.

## Data Availability

The MLST allelic profile and sequence for isolate AAUH 190,263 is available at https://pubmlst.org/ Additional WGS-data is available on specific request to the corresponding Author.

## Abbreviations

AST: Antibiotic susceptibility testing
MIC: Minimum inhibitory concentration
MLST: Multi-locus sequence typing
PFGE: Pulse-field gel electrophoresis
ST: Sequence type
SNP: Single nucleotide polymorphism
TTD: Time to detection
